# Ideal Cardiovascular Health Metrics Are Associated with Disability Independently of Vascular Conditions

**DOI:** 10.1371/journal.pone.0150282

**Published:** 2016-02-29

**Authors:** Saravana Devulapalli, Hazem Shoirah, Mandip S. Dhamoon

**Affiliations:** Department of Neurology, Icahn School of Medicine at Mount Sinai, New York, New York, United States of America; The University of Tokyo, JAPAN

## Abstract

**Background:**

Vascular risk factors may be associated with disability independently of vascular events. We examined whether the American Heart Association’s 7 ideal cardiovascular health (CVH) metrics were independently associated with disability in a nationally representative cohort.

**Methods:**

Adults age ≥20 years from the National Health and Nutrition Examination Survey 2005–2012 were included. Ideal CVH was calculated as a composite of 7 measures, each scored 0–2. Primary predictors were number of ideal CVH metrics and score of CVH metrics. The outcome was a dichotomous score from 20 activities of daily living (ADL) and instrumental ADLs. Unadjusted and adjusted weighted logistic models estimated associations between ideal CVH and disability. The data were analyzed in 2015.

**Results:**

Among 22692 participants, mean age was 46.9 years. Cardiac disease and stroke were present in 6.6% and 2.8%; 90.3% had poor physical activity and 89.9% poor diet. Among 3975 individuals with full CVH data, in fully adjusted models, OR for disability was 0.90 (95% CI 0.83–0.98) per point increase in ideal CVH score, and 0.84 (0.73–0.97) per additional number of ideal CVH metrics.

**Conclusions:**

CVH metrics were strongly and significantly associated with reduced odds of disability independently of vascular and non-vascular conditions. Poorer CVH may cause subclinical vascular disease resulting in disability.

## Introduction

Individuals with disability may have limitations in activities of daily living, instrumental activities of daily living, social activities and leisure activities [1]. Cardiovascular disease is the third most common cause of disability in the U.S., after arthritis and back problems [2]. Functional limitations from stroke and cerebral dysfunction comprise a large proportion of this burden [3, 4]. Although non-vascular disorders such as neuropathy, pain, blindness, lung diseases and mental illness contribute to disability, cardiovascular disease is the leading cause of morbidity and mortality in the U.S. [2, 5] Furthermore, disability from non-vascular etiologies can be a risk factor for subsequent vascular disease [6]. Because of the high prevalence of disability and the complexity of its management, it is important to capture the modifiable predictors and correlates of disability with easily measured variables.

The American Heart Association (AHA) has successfully promoted medically oriented interventions for cardiovascular disease in the past. However, it is evident that health is a broader construct than just the absence of clinically evident disease. The AHA introduced the concept of ideal cardiovascular health (CVH) with the goal of improving the CVH of all Americans by 20%, while reducing deaths from coronary vascular disease and stroke by 20% by 2020 [5]. The AHA's ‘Strategic Impact Goal Through 2020 and Beyond’ defined ideal, intermediate or poor CVH based on smoking status, physical activity, diet, weight, cholesterol, blood pressure and fasting blood glucose [5].

In previous studies, ideal CVH has been found to be associated with lower aortic intima-media thickness, greater aortic elasticity, favorable profiles of circulating cardiovascular disease biomarkers, and a lower prevalence of subclinical cardiovascular disease [7]. Ideal CVH has been also shown to be a strong predictor of all-cause mortality [8]. Factors determining CVH appear to be associated with non-cardiovascular outcomes as well, such as cognition [3, 4]. These findings have provided support for CVH as a valid construct [9].

Few prior studies have examined the relationship between ideal CVH and disability, and none has done so in a nationally representative population [10]. We sought to examine this relationship in the National Health and Nutrition Examination Survey (NHANES) study, and we hypothesized that: 1) there is an association between ideal CVH metrics and different measures of disability, 2) this association is independent of clinical cardiovascular events, and 3) the predictive ability of the ideal CVH metrics is different among those with cardiovascular disease compared to those without.

## Methods

NHANES is a large, nationally representative cross-sectional survey conducted in 2-year cycles in the U.S., focusing on health conditions and behaviors, diet, physical examination findings, and laboratory results. The sampling methodology seeks to create nationally representative estimates for the non-military, noninstitutionalized U.S. population using a complex, multistage probability design that samples individuals in strata defined by geographical location and race-ethnicity. Evaluations included interviews, physical examinations, and laboratory tests on blood samples.

The design and methods of NHANES have been described elsewhere [11]. Written informed consent was obtained from all participants by the NHANES study, and the study was reviewed by the CDC/National Center for Health Statistics. For this analysis, all data were obtained from 4 2-year NHANES survey cycles from 2005 to 2012 in individuals 20 years and older. The respondents’ actual or imputed date of birth was used to calculate age. Gender and marital status were obtained from standardized interviews. Race-ethnicity was self-reported and classified as non-Hispanic Black, non-Hispanic White, and Mexican Hispanic, and other Hispanic. Education status was assessed by asking the highest grade or level of school completed or the highest degree received, and education was dichotomized as having a high school education or less. Individuals were considered as having health insurance if they had a health care plan obtained through employment or purchased directly as well as government programs such as Medicare and Medicaid.

Socioeconomic status was defined by the poverty income ratio, which was calculated by dividing household income by the U.S. federal poverty threshold specific to family size and year. The amount of alcohol intake per day was assessed using standardized questionnaires assessing daily intake of various beverages including liquor, beer, wine, wine coolers, and others. History of coronary heart disease, myocardial infarction, congestive heart failure, angina pectoris, arthritis, asthma, cancer or malignancy, chronic bronchitis, gout, emphysema, liver conditions, stroke, and thyroid problems was obtained by self-report of a physician or other health professional diagnosis of that particular condition. Vision impairment was defined as having trouble seeing, even when wearing glasses or contact lenses. Abnormal mental health included having stress, depression, and problems with emotions over the past 30 days.

Smoking status was obtained with a standardized questionnaire. Body mass index (BMI) was calculated as kg of weight divided by height in meters squared, both obtained during the standardized examination. Total cholesterol level and fasting glucose level measurement have been previously described, and blood pressure was measured according to a standard protocol [11]. The use of anti-hypertensive, diabetes, and cholesterol-modifying medications was assessed by self-report. Two interviewer-administered 24-hour recall questionnaires were used to assess dietary intake. The AHA ideal CVH definition of a healthy diet includes ≥4.5 cups per day of fruits and vegetables, more than two 3.5-ounce servings per week of fish, more than three 1-ounce servings per day of whole grains, <1500 mg/d of sodium, and <450 kcal/wk of added sugar in sugar-sweetened beverages. The MyPyramid Equivalents Database was used to calculate nutrition components of individuals’ food intake using methodology established by the USDA Center for Nutrition Policy and Promotion. Physical activity was assessed by self-report using questionnaires that quantified the frequency and duration of specific moderate-intensity activities and vigorous-intensity activities over the prior week or month. Physical activity related to transportation and household activities were also assessed.

The seven CVH factors were classified into ideal, intermediate or poor categories as outlined in Table 1 following AHA/American Stroke Association definitions. CVH was analyzed according to 2 definitions. Participants were classified by number of ideal CVH metrics present at baseline (0 to 7) for “number of ideal CVH metrics." For “score of CVH metrics,” 0 was given for a category of “poor,” 1 was given for “intermediate,” and 2 for “ideal” for each CVH factor. The total score (with a possible range 0 to 14) for each individual was calculated by summing values for each of the 7 CVH metrics. For both number of ideal CVH metrics and score of CVH metrics, higher values reflect better CVH.

**Table 1:** Definitions of the 7 ideal cardiovascular health factors.

Cardiovascular Health indicator	Status of indicator		
	Poor	Intermediate	Ideal
Smoking	Current	Quit ≤1 year	Never or quit >1 year
Body mass index	≥30 kg/m^2^	25 to <30 kg/m^2^	<25 kg/m^2^
Physical activity	No moderate or vigorous activity	1–149 min/wk moderate intensity, 1–74 min/wk vigorous intensity, or equivalent combination	≥150 min/wk moderate intensity,≥75 min/wk vigorous intensity, or equivalent combination
Diet*	0–1 healthy components	2–3 healthy components	4–5 healthy components
Total cholesterol	≥240 mg/dL	Treated to <200 mg/dL or 200–239 mg/dL	Untreated and <200 mg/dL
Blood pressure	≥140/90 mmHg	Treated to <120/<80 mmHg or 120–139/80–89 mmHg	Untreated and <120/<80 mmHg
Fasting plasma glucose	≥126 mg/dL	Treated to <100 mg/dL or 100–125/mg/dL	Untreated and <100 mg/dL

*based on 5 health dietary metrics: (1) ≥4.5 cups of fruits and vegetables/day, (2) >2 3.5-oz servings of fish/week, (3) >3 1-oz-equivalent servings of fiber-rich whole grains/day, (4) <1500 mg sodium/day, and (5) ≤450 kcal sugar-sweetened beverages/week)

The primary outcome was disability based upon the physical functioning questionnaire, which provides self-reported data on functional limitations caused by long-term physical, mental, and emotional problems or illness. Individuals aged 20 years and older were asked 20 questions that assessed ability to perform activities of daily living (ADLs), instrumental activities of daily living (IADLs), lower extremity mobility, general mobility, and social and leisure activities (S1 Table). Responses were coded as: no difficulty, some difficulty, much difficulty, or inability to perform the activity. Physical disability was defined as having any level of difficulty in response to any of the 20 questions.

## Statistical Analysis

Due to the complex, multistage sampling used in NHANES, specific procedures must be used that incorporate the stratum, cluster, and weighting of data from each individual in order to accurately estimate associations and variances. We followed the specifications and recommendations for analysis and weighting outlined on the NHANES website [12]. Specifically, among the available weights for each survey component, the weight of each individual was chosen based upon the selection group of the most restrictive variable. Because 4 survey cycles of the continuous NHANES survey were combined, each weight was divided by 4.

Distributions of variables were calculated using the procedure surveymeans (SAS version 9.4, Cary, NC), and results were summarized with means for continuous variables (with 95% confidence intervals [CI]) or proportions for categorical variables (with 95% CI). The distributions of poor, intermediate, and ideal values for each of the 7 ideal CVH indicators were calculated.

Because the distribution of disability scores was highly skewed, with the majority of participants having no disability, the outcome of disability was dichotomized into no disability (disability score of 0) vs. any disability (disability score of > = 1), and the procedure surveylogistic was used to estimate the probability of any disability and provide odds ratios and 95% CI for each covariate. The primary predictors, ideal CVH score and number of ideal CVH metrics, were tested in separate models. Only those with complete data on ideal CVH factors were included in these models. Groups of covariates were added successively in a pre-specified manner: demographics (age, sex, race-ethnicity), social factors (marital status, health insurance status, education), medical conditions (history of cardiac disease, arthritis, asthma, cancer, bronchitis, emphysema, liver disease, stroke, and thyroid disease), and visual impairment and socioeconomic status. After the addition of each successive group of covariates, variables not significantly associated with the outcome (at a p-value of <0.15) were removed from the model.

In order to assess whether the associations between the primary predictors and disability differed by values of covariates, we tested interactions between each covariate and the primary predictors (ideal CVH score and number of ideal CVH metrics) in fully adjusted models. For significant interaction terms, we presented odds ratios and 95% CI for disability for each primary predictor in strata (present and absent) of the interaction term.

Finally, in order to further explore associations between the primary predictors and different measures of disability, the 20 items that constituted the composite disability outcome were tested as separate outcomes as dichotomous variables (0 versus 1) in fully adjusted multivariable models, and the ORs and 95% CIs were summarized in a forest plot using the RMETA package of R (version 3.1.2). The data were analyzed in 2015.

## Results

The total NHANES cohort over 4 cycles included 22692 participants with a mean age of 46.9 years. Distributions of demographics, medical conditions, and ideal CVH indicators are shown in Table 2. The population was predominantly non-Hispanic White; the majority had at least high school education and were covered by insurance. The mean poverty income ratio was 2.99, which is above the poverty level. The vascular conditions of cardiac disease and stroke were present in 6.6% and 2.8% of the population, respectively. The most common non-vascular conditions were arthritis (24.1%) and vision abnormality (16.5%).

**Table 2:** Baseline demographics of study population*.

Variable			N	Mean or proportion (%)	95% CI lower limit	95% CI upper limit
Age			22692	46.9	46.3	47.6
Male			11043	48.1%	47.4	48.7
Non-Hispanic Black			4927	11.4%	9.6	13.2
Non-Hispanic White			10273	68.8%	65.4	72.2
Mexican-American			3716	8.1%	6.5	9.8
Other Hispanic			2030	5.0%	3.8	6.2
Married			11672	59.2%	57.5	60.8
At least high school education			16293	81.5%	80.0	82.9
Covered by insurance			17311	80.3%	79.0	81.6
History of cardiac disease			1957	6.6%	6.1	7.1
History of stroke			907	2.8%	2.5	3.2
History of arthritis			6041	24.1%	23.0	25.3
History of asthma			3081	14.0%	13.2	14.9
History of cancer			2098	9.2%	8.6	9.8
History of bronchitis			1273	5.7%	5.1	6.3
History of emphysema			499	1.9%	1.6	2.1
History of liver disease			797	3.1%	2.8	3.4
History of thyroid disease			2132	10.0%	9.4	10.7
History of vision abnormality			4439	16.5%	15.7	17.4
Abnormal mental health days in past 30 days			19612	3.9	3.7	4.1
Poverty-income ratio			20758	2.99	2.92	3.07
Number of alcoholic drinks per day			12922	0.71	0.68	0.75
Ideal cardiovascular health indicators:						
Smoking:						
	Poor		4829	21.7%	20.5	22.7
	Intermediate		638	3.1%	2.7	3.4
	Ideal		17143	75.2%	74.1	76.2
Body mass index:						
	Poor		7854	34.7%	33.5	35.9
	Intermediate		7248	33.6%	32.5	34.6
	Ideal		6416	31.7%	30.3	32.9
Diet:						
	Poor		16142	89.95%	89.1	90.7
	Intermediate		1936	9.98%	9.1	10.7
	Ideal		15	0.07%	0.02	0.11
Total cholesterol:						
	Poor		1253	13.7%	12.7	14.5
	Intermediate		3981	41.8%	40.3	43.1
	Ideal		3967	44.5%	42.9	46.1
Blood pressure:						
	Poor		611	4.4%	3.9	4.9
	Intermediate		2389	19%	17.6	20.1
	Ideal		8366	76.6%	75.2	78.0
Glucose:						
	Poor		1104	8.7%	7.8	9.6
	Intermediate		3456	38%	36.2	39.6
	Ideal		4452	53.3%	51.3	55.2
Physical activity:						
	Poor		20789	90.3%	89.4	91.1
	Intermediate		74	0.4%	0.2	0.6
	Ideal		1829	9.3%	8.4	10.0
Number of ideal CVH metrics present			3975	3.12	3.05	3.20
Ideal CVH score			3975	7.6	7.5	7.7

*Note: The ‘N’ column for continuous variables refers to the number of individuals with available data; for categorical variable, it refers to the number of individuals with a positive value of the attribute listed

In terms of CVH indicators, a markedly high proportion of the population had poor physical activity (90.3%) and diet (89.9%). Among the 3975 individuals with full data on all 7 ideal CVH metrics, the mean number of ideal CVH metrics was 3.12 (95% CI 3.05–3.20; S1 Fig) and ideal CVH score was 7.6 (7.5–7.7; S2 Fig). Among those with full data on disability measures as well as all CVH components, 87.8% had no disability and 12.2% had some disability.

As Table 3 shows, both ideal CVH score and number of ideal CVH metrics were strongly and significantly associated with reduced odds of disability, in unadjusted models as well as after adjustment for demographics, social factors, medical conditions, and visual impairment and SES. In fully adjusted models, the OR for disability was 0.90 (95% CI 0.83–0.98) per point increase in ideal CVH score, and the OR for disability was 0.84 (0.73–0.97) per point increase in number of ideal CVH metrics.

**Table 3:** The association between ideal cardiovascular indicators and disability in unadjusted and adjusted models^†^.

Variable	Odds ratio	95% CI	p-value
**Ideal CVH score:**			
Unadjusted model	0.76	0.71–0.81	< .0001
Adjusted for demographics*	0.80	0.74–0.85	< .0001
Adjusted for social factors**	0.82	0.76–0.88	< .0001
Adjusted for medical conditions***	0.87	0.81–0.93	0.0003
Adjusted for visual impairment and socioeconomic status ‡	0.90	0.83–0.98	0.0194
**Number of ideal CVH metrics:**			
Unadjusted model	0.65	0.58–0.73	< .0001
Adjusted for demographics*	0.72	0.64–0.82	< .0001
Adjusted for social factors**	0.75	0.66–0.85	< .0001
Adjusted for medical conditions***	0.82	0.72–0.93	0.0028
Adjusted for visual impairment and socioeconomic status‡	0.84	0.73–0.97	0.0225

†CVH = cardiovascular health

*adjusted for age and male sex

**adjusted for age, marital status, and education

***adjusted for age, marital status, education, cardiac disease, arthritis, asthma, bronchitis, and stroke

‡adjusted for age, marital status, education, cardiac disease, arthritis, asthma, bronchitis, and stroke

Out of all covariates tested, there were significant interactions with ideal CVH measures only for cardiac disease (p-value for interaction = 0.007 for ideal CVH score and 0.01 for number of ideal CVH indicators) and stroke (p-value for interaction 0.12 and 0.11 respectively; Table 4). Among those with cardiac disease and stroke, there was a paradoxical trend for increased odds of disability for increasing values of both ideal CVH score and number of ideal CVH indicators.

**Table 4:** Interactions between ideal cardiovascular health indicators and covariates*.

Variable (p-value for interaction)	Stratum	Odds ratio for disability†	95% CI	p-value
**Predictor: Ideal CVH score:**				
Cardiac disease (p = 0.007)	Absent	0.87	0.79–0.95	0.003
	Present	1.19	0.93–1.52	0.16
Stroke (p = 0.12)	Absent	0.89	0.82–0.97	0.01
	Present	2.12	0.93–4.85	0.07
**Predictor: number of ideal CVH indicators:**				
Cardiac disease (p = 0.01)	Absent	0.79	0.68–0.92	0.003
	Present	1.73	1.05–2.85	0.03
Stroke (p = 0.11)	Absent	0.84	0.72–0.97	0.02
	Present	1.54	0.63–3.78	0.3

†CVH = cardiovascular health

*Models are adjusted for: age, marital status, arthritis, asthma, stroke, visual impairment, socioeconomic status, and cardiac disease–minus the stratifying variable

S3 and S4 Figs show forest plots of the odds ratios for associations between number of ideal cardiovascular indicators and each disability item (S1 Table). For the majority of disability items, there was an inverse relationship with ideal CVH metrics, in parallel with the findings for the composite disability variable shown above. For both ideal CVH indicators (S3 and S4 Figs), only Rooms, Fork, and Leisure had OR close to or above 1.

## Discussion

In this cross-sectional analysis in a large, nationally representative population, we found that ideal CVH score and number of ideal CVH metrics were consistently and strongly associated with reduced odds of disability. This effect was seen in fully adjusted models and was independent of medical and vascular conditions such as stroke and MI. In fully adjusted models, the magnitude of association was such that for each point increase in ideal CVH score, there was a 10% reduced odds of disability, and for each additional ideal CVH metric, there was a 16% reduced odds of disability. CVH metrics were associated relatively consistently with individual ADL and IADL components of disability. There was also a significant interaction with both stroke and MI, such that ideal CVH score and number of ideal CVH metrics were associated with disability only in those without stroke or MI. In terms of distribution of CVH metrics, approximately half of the cohort had 3–4 ideal CVH metrics, similar to findings from prior, smaller studies [7]. In terms of ideal CVH components, there were high proportions of the cohort who had poor CVH status for physical activity (90.3%) and diet (90.0%). BMI was evenly distributed among poor, intermediate, and ideal categories.

To our knowledge, this is only the second study of the association between ideal CVH metrics and disability. In a prospective analysis of functional status in the multiethnic Northern Manhattan Study [10], the number of ideal CVH categories and higher CVH metric scores were associated with long-term functional status, even after adjustment for stroke and MI. However, this cohort was not representative of the U.S. population in terms of race-ethnicity and distributions of age and vascular risk factors. The current NHANES study demonstrates a strong and consistent cross-sectional association between ideal CVH and reduced odds of disability that can be inferred to exist broadly in the U.S. population. In addition, in this analysis, we were able to adjust for arthritis and visual impairment, two large contributors to disability. The substantive effect sizes for the effect of ideal CVH on disability were independent of the effects of arthritis and visual impairment on disability.

Several mechanisms may underlie the relationship between CVH and disability in the absence of clinical vascular disease. Vascular risk factors, which are represented by ideal CVH components, may lead not only to clinical events but also to an accumulation of subclinical vascular disease resulting in disability prior to the onset of clinically apparent events. Subclinical vascular disease includes subclinical infarcts [13, 14], subclinical coronary disease, white matter disease [15–18], and vascular dysfunction [19]. Subclinical vascular disease has an impact on gait, cognition, activity tolerance, and autonomic functions, suggesting that the disability seen in this study may be a result of vascular functional impairment [10]. Of note, in this analysis, ideal CVH metrics were not predictive of disability among those with stroke or MI, suggesting that once an individual has a clinical vascular event, the vascular risk factors summarized in CVH metrics are less useful as predictors of additional disability.

There are several strengths of this study. NHANES is a large, nationally representative sample with respect to age, gender, race, level of education, socio-economic status, and vascular risk factors. Disability was thoroughly investigated through a comprehensive questionnaire that covered all domains of functionality including and extending beyond the domains that are evaluated by Barthel Index and Duke Activity Status Index, 2 commonly used measures of disability. Our analysis fully adjusted for non-vascular causes of disability, allowing us to postulate vascular mechanisms as an underlying mediator between CVH and disability. The major limitation of this analysis was that it was cross-sectional, limiting our ability to establish a causal relationship between CVH factors and subsequent disability. Also, although the population studied was large, full data on ideal CVH metrics was available for a smaller number of subjects.

The AHA’s 2020 Strategic Impact Goals are set to reduce the burden of cardiovascular disease (CVD) by 20% and increase CVH by 20% by the year 2020. This study, along with prior publications, demonstrates the importance of promoting ideal cardiovascular health as a national strategy, not just for the reduction of CVD, but also for its favorable impact on disability. It is reasonable to expect a parallel decrease in disability and an improvement in population functional status beyond the targeted 20% reduction of CVD.

## Supporting Information

S1 Fig Histogram of values for number of ideal CVH indicators. (DOCX)

S2 Fig Histogram of values for score of ideal CVH metrics. (DOCX)

S3 Fig Forest plot showing associations between number of ideal cardiovascular indicators and each disability item. (DOCX)

S4 Fig Forest plot showing associations between score of ideal cardiovascular indicators and each disability item. (DOCX)

S1 Table Abbreviations of the 20 disability questions asked of participants. (DOCX)
