# Supplementary material for: Ideal Cardiovascular Health Metrics Are Associated with Disability Independently of Vascular Conditions
Source: PLoS One. 2016 Feb 29;11(2):e0150282. doi: 10.1371/journal.pone.0150282 (PMC4771828; doi:10.1371/journal.pone.0150282)
Supplement: S1 Fig — (DOCX) [file pone.0150282.s001.docx]

**S1 Fig. Histogram of values for number of ideal CVH indicators**
