# Supplementary material for: Ideal Cardiovascular Health Metrics Are Associated with Disability Independently of Vascular Conditions
Source: PLoS One. 2016 Feb 29;11(2):e0150282. doi: 10.1371/journal.pone.0150282 (PMC4771828; doi:10.1371/journal.pone.0150282)
Supplement: S2 Fig — (DOCX) [file pone.0150282.s002.docx]

**S2 Fig. Histogram of values for score of ideal CVH metrics**
