# Supplementary material for: Ideal Cardiovascular Health Metrics Are Associated with Disability Independently of Vascular Conditions
Source: PLoS One. 2016 Feb 29;11(2):e0150282. doi: 10.1371/journal.pone.0150282 (PMC4771828; doi:10.1371/journal.pone.0150282)
Supplement: S3 Fig — (DOCX) [file pone.0150282.s003.docx]

**S3 Fig. Forest plot showing associations between number of ideal cardiovascular indicators and each disability item**


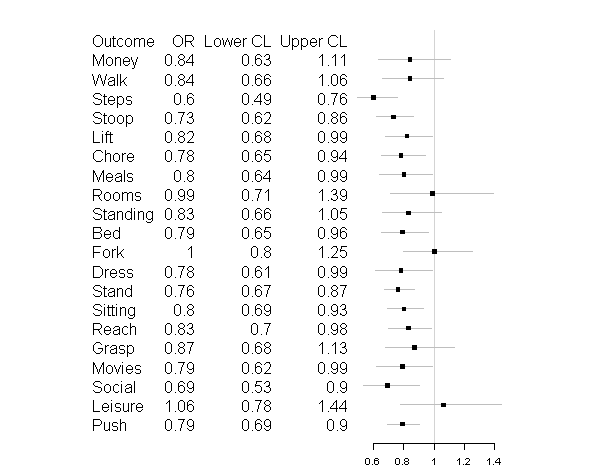
 ^3, 5^

Note: Each line represents a separate outcome, with main predictor number of ideal cardiovascular health indicators, also adjusted for: age, marital status, history of cardiac disease, history of arthritis, history of asthma, history of stroke, visual difficulty, and socioeconomic status. Definitions of each outcome are in the text.
