# Supplementary material for: Ideal Cardiovascular Health Metrics Are Associated with Disability Independently of Vascular Conditions
Source: PLoS One. 2016 Feb 29;11(2):e0150282. doi: 10.1371/journal.pone.0150282 (PMC4771828; doi:10.1371/journal.pone.0150282)
Supplement: S1 Table — (DOCX) [file pone.0150282.s005.docx]

**S1 Table: Abbreviations of the 20 disability questions asked of participants**

| Outcome Variable Name | Disability question asked of the participant:  “The next questions ask about difficulties [you/survey person] may have doing certain activities because of a health problem. By "health problem" we mean any long-term physical, mental or emotional problem or illness [not including pregnancy].  By [yourself/himself/herself] and without using any special equipment, how much difficulty [do you/does survey person] have . . .” |
| --- | --- |
| Money | Managing [your/his/her] money [such as keeping track of  [your/his/her] expenses or paying bills]? |
| Walk | Walking for a quarter of a mile [that is about 2 or 3 blocks]? |
| Steps | Walking up 10 steps without resting? |
| Stoop | Stooping, crouching, or kneeling? |
| Lift | Lifting or carrying something as heavy as 10 pounds [like a  sack of potatoes or rice]? |
| Chore | Doing chores around the house [like vacuuming, sweeping,  dusting, or straightening up]? |
| Meals | Preparing [your/his/her] own meals? |
| Rooms | Walking from one room to another on the same level? |
| Standing | Standing up from an armless straight chair? |
| Bed | Getting in or out of bed? |
| Fork | Eating, like holding a fork, cutting food or drinking from a glass? |
| Dress | Dressing [yourself/himself/herself], including tying shoes,  working zippers, and doing buttons? |
| Stand | Standing or being on [your/his/her] feet for about 2 hours? |
| Sitting | Sitting for about 2 hours? |
| Reach | Reaching up over [your/his/her] head? |
| Grasp | Using [your/his/her] fingers to grasp or handle small objects? |
| Movies | Going out to things like shopping, movies, or sporting events? |
| Social | Participating in social activities [visiting friends, attending  clubs or meetings or going to parties]? |
| Leisure | Doing things to relax at home or for leisure [reading, watching  TV, sewing, listening to music]? |
| Push | Pushing or pulling large objects like a living room chair? |
